# Unacceptable treatment outcomes and associated factors among India's initial cohorts of multidrug-resistant tuberculosis (MDR-TB) patients under the revised national TB control programme (2007–2011): Evidence leading to policy enhancement

**DOI:** 10.1371/journal.pone.0193903

**Published:** 2018-04-11

**Authors:** Malik M. Parmar, Kuldeep Singh Sachdeva, Puneet K. Dewan, Kiran Rade, Sreenivas A. Nair, Rashmi Pant, Sunil D. Khaparde

**Affiliations:** 1 World Health Organization–Country Office for India, New Delhi, India; 2 National AIDS Control Organization, Ministry of Health and Family Welfare, Government of India, New Delhi, India; 3 Bill & Melinda Gates Foundation, India Country Office, New Delhi, India; 4 Public Health Foundation of India, Hyderabad, India; 5 Central TB Division, Ministry of Health and Family Welfare, Government of India, New Delhi, India; Institut de Pharmacologie et de Biologie Structurale, FRANCE

## Abstract

**Background:**

Globally, India has the world’s highest burden of multidrug-resistant tuberculosis (MDR-TB). Programmatic Management of Drug Resistant TB (PMDT) in India began in 2007 and nationwide coverage was achieved in early 2013. Poor initial microbiological outcomes under the Revised National Tuberculosis Control Programme (RNTCP) prompted detailed analysis. This is the first study on factors significantly associated with poor outcomes in MDR-TB patients treated under the RNTCP.

**Objective:**

To evaluate initial sputum culture conversion, culture reversion and final treatment outcomes among MDR-TB patients registered in India from 2007 to early 2011 who were treated with a standard 24-month regimen under daily-observed treatment.

**Methods:**

This is a retrospective cohort study. Clinical and microbiological data were abstracted from PMDT records. Initial sputum culture conversion, culture reversion and treatment outcomes were defined by country adaptation of the standard WHO definitions (2008). Cox proportional hazards modeling with logistic regression, multinomial logistic regression and adjusted odds ratio was used to evaluate factors associated with interim and final outcomes respectively, controlling for demographic and clinical characteristics.

**Results:**

In the cohort of 3712 MDR-TB patients, 2735 (73.6%) had initial sputum culture conversion at 100 median days (IQR 92–125), of which 506 (18.5%) had culture reversion at 279 median days (IQR 202–381). Treatment outcomes were available for 2264 (60.9%) patients while 1448 (39.0%) patients were still on treatment or yet to have a definite outcome at the time of analysis. Of 2264 patients, 781 (34.5%) had treatment success, 644 (28.4%) died, 670 (29.6%) were lost to follow up, 169 (7.5%) experienced treatment failure or were changed to XDR-TB treatment. Factors significantly associated with either culture non-conversion, culture reversion and/or unfavorable treatment outcomes were baseline BMI < 18; ≥ seven missed doses in intensive phase (IP) and continuation phase (CP); cavitary disease; prior treatment episodes characterized by re-treatment regimen taken twice, longer duration and more episodes of treatment; any weight loss during treatment; males and additional resistance to first line drugs (Ethambutol, Streptomycin). In a subgroup of 104 MDR-TB patients, 62 (59.6%) had Ofloxacin resistance among whom only 25.8% had treatment success, half of the success (54.8%) seen in Ofloxacin sensitive patients. Baseline susceptibility to Ofloxacin (HR 2.04) and Kanamycin (HR 4.55) significantly doubled and quadrupled the chances for culture conversion respectively while baseline susceptibility to Ofloxacin (AOR 0.37) also significantly reduced the odds of unfavorable treatment outcomes (p value ≤0.05) in multinomial logistic regression model.

**Conclusion:**

India’s initial MDR-TB patients’ cohort treated under the RNTCP experienced poor treatment outcomes. To address the factors associated with poor treatment outcomes revealed in our study, a systematic multi-pronged approach would be needed. A series of policies and interventions have been developed to address these factors to improve DR-TB treatment outcomes and are being scaled-up in India.

## Background

India has the highest burden of multi-drug resistant tuberculosis (MDR-TB) with an estimated 84,000 MDR-TB patients emerging in 2016 amongst the notified pulmonary TB patients [1]. An equal volume of pulmonary TB patients are expected to be treated in the private sector and not notified under the national programme [2], including an indirect estimate of 63,000 drug resistant TB patients derived from 147,000 incident MDR-TB estimated to emerge in India [1]. In 2007, under the Revised National TB Control Programme (RNTCP), India introduced programmatic management of drug resistant TB (PMDT) erstwhile the DOTS Plus programme when diagnosis was offered initially only to those experiencing treatment failures among previously treated patients. Since March 2013, PMDT services are available to a wider group of presumptive MDR-TB patients across the country [3].

Until March 2011, PMDT services were available in 150 districts of 15 states covering a population of 331.5 million (30.1%) of India [4]. For diagnosis of MDR-TB, drug susceptibility testing (DST) on transported sputum samples was offered mainly to TB patients with treatment failure to the first line regimen, previously treated patients with non-conversion on follow up smear microscopy and contacts of known MDR-TB patients using phenotypic methods (solid/liquid culture drug susceptibility test [DST]) for first line drugs in 27 laboratories in India. Genotypic DST with Line Probe Assay (LPA) was initiated geographically at four laboratories over the course of the study period with the same enrolment criteria [4,5). Laboratory confirmed MDR-TB (including Rifampicin Resistant [RR-TB]) patients were initiated on a standardized treatment regimen for MDR-TB with six to nine months of intensive phase (IP) with Kanamycin [Km], Ofloxacin [Ofx], Ethonamide [Eto], Cycloserine [Cs], Pyrazinamide [Z] and Ethambutol [E] followed by 18 months of continuation phase (CP) with Km and Z discontinued after IP [5]. Para amino salicylic acid (PAS) Sodium was used as a substitute drug in case of intolerance to any of these drugs [5]. Treatment was offered through 24 airborne infection control compliant drug resistant TB centers (DR-TB centers), erstwhile the DOTS Plus sites [4,5). Initial hospitalization for expert consultation was followed by ambulatory treatment of the MDR-TB patients directly observed by a health worker or community volunteer using monthly patient boxes supplied to the provider to ensure uninterrupted drug supply [5].

Poor initial microbiological outcomes observed in the initial PMDT pilot cohorts of the RNTCP from 2007–08 prompted this detailed analysis. The magnitude of patients to be managed in India also makes it critical that the treatment outcomes of MDR-TB patients and the factors affecting them are systematically evaluated to guide the national programme to take informed decisions on policies and strategies to improve treatment outcomes of subsequent cohorts of patients.

This study was conducted to evaluate microbiological and treatment outcomes along with risk factors for poor outcomes, among the initial cohort of all laboratory-confirmed MDR-TB patients initiated on treatment and registered under RNTCP PMDT services from August 2007 to March 2011. We also report how the results from this analysis influenced substantial policy changes for PMDT in India.

## Methods

### Study design

This study is a retrospective cohort analysis based on RNTCP PMDT records.

### Study setting

In India, as of March 2011, RNTCP PMDT services were available in 150 districts of 15 states covering a population of 331.5 million [4,5). A total of 4217 lab-confirmed MDR-TB patients (including RR-TB patients) were diagnosed through 27 laboratories certified by national reference laboratory for first line DST and registered under the RNTCP for treatment through 24 DR-TB centers.

### Study population and sampling

The study population included all MDR-TB and RR-TB patients consecutively registered for treatment from August 2007 to March 2011 at the initial 15 DR-TB Centers under RNTCP PMDT services. These 15 DR-TB centers catered to a population of 227 million residing in 108 districts of seven states of India. Data collection began in March 2012 and was closed on 30^th^ June 2012. There were no predefined exclusion criteria for enrollment in the study, and we enrolled the entire eligible population. In the cohort of patients included in this analysis, drug susceptibility tests were offered to a highly selected group of patients that included TB patients who were either treatment failures of first line regimen or previously treated patients with non-conversion on follow up smear microscopy or contacts of known MDR-TB patients using phenotypic methods (solid Lowenstein Jensen [LJ] media) in majority of patients. First line LPA was available only in four centralized labs in the later part of the study period. Standard WHO definitions (2008) were used to define MDR-TB, initial culture conversion, culture reversion and final treatment outcomes as reflected in the 2010 version of the RNTCP DOTS Plus guidelines.[5,6]

### Study variables

The independent variables considered in the models were basic demographic characteristics; body mass index (BMI); type of patient during registration of initial treatment; treatment experience in terms of exposure to re-treatment regimen taken twice, number, duration and source of prior treatment whether government, private or other public sector facilities; clinical and microbiological data like co-morbidities (HIV and Diabetes) taken from patients’ baseline pre-treatment evaluation; resistance patterns to all first line drugs (Streptomycin [S], Isoniazid [H], Rifampicin [R], Ethambutol [E]); grades of chest radiographs and cavitation on chest radiographs [7]; treatment delay by DST method; weight change at 6 and 12 months against baseline; poor treatment adherence defined using ≥7 missed doses during IP and CP as a dichotomous classification similar to those used in previous studies [8,9]. To examine the validity of cut-offs for missed doses in IP and CP, we used a sharp regression discontinuity analysis [10]. The density curves for these variables were examined to identify the presence of discontinuity and explore the effect of different cut-offs on culture positivity estimates in the survival analysis. For a subgroup of patients, baseline second-line DST results for Ofx, Km and Eto were available and considered for analysis.

The dependent variables studied for all MDR-TB patients included initial culture conversion defined as two consecutive negative culture results more than 30 days apart, culture reversion defined as two consecutive positive culture results more than 30 days apart after conversion and, final treatment outcomes classified as favorable (cured, treatment completed) and unfavorable (deaths, lost to follow up [LTFUs] and treatment failure) using standard WHO definitions (2008) [5,6]. Additional outcome definitions in the erstwhile RNTCP DOTS Plus Guidelines (2010) like switched to XDR-TB regimen and still on treatment beyond 24–27 months depending on monthly extension of IP up to a maximum of 9 months were counted in treatment failures while those whose treatment was stopped due to adverse drug reaction or other medical conditions were counted in LTFU to align with the standard WHO definitions of MDR-TB treatment outcomes. Patients in whom the data on the above variable were not available were placed in unknown category.

### Data collection

Data on clinical and microbiological variables including the interim and final treatment outcomes of MDR-TB patients registered under RNTCP PMDT services until 31^st^ March 2011 in the 15 selected DR-TB centers were systematically collected and analyzed in this study. The remaining nine DR-TB centers were not included in the study as PMDT services had commenced there in early 2011 and hence, the data required for analysis of earliest interim outcomes were not available as most of the patients enrolled on treatment in these centers were very early in their treatment course. The data were extracted from the existing paper based RNTCP PMDT programme records maintained at each of the 15 selected DR-TB centers viz. the treatment registers, treatment cards and drug-o-gram that detailed history of exposure to past treatment episodes. The chest radiographs were graded into mild, moderately advanced and far advanced as well as cavitary or non-cavitary by an expert panel of physicians at the DR-TB centers using standard reference [7]. In 2007, a single early pilot site at Gujarat state was linked for drug susceptibility testing at National Institute for Research in Tuberculosis—Chennai, a supranational reference laboratory for a few months. Results of SL-DST along with FL-DST were available for a consecutively enrolled small number of patients that was used for a subgroup analysis in this study.

Data were entered in pre-structured, protected spreadsheets in MS Excel (Microsoft Corporation, Redmond, WA, USA) (S1 Data) by trained team of data entry operators and statistical assistants under supervision of the Medical Officers at DR-TB Centers and WHO RNTCP Medical Consultants. Validation of the entire data set on completeness and internal consistency with re-validation of discrepancies if any was undertaken by WHO RNTCP National Consultants. Random on-site validation of approximately 25% of the data was also done during visits to selected DR-TB centers. Minimal issues on completeness, correctness and consistency of data including correct interpretation of outcomes identified during the validation exercise were corrected in the master data before analysis.

### Statistical analysis

Data analysis was performed using Stata Software version 12.1 (StataCorp LP, College Station, TX, USA). Survival analysis was used to determine risk factors for time to culture conversion and reversion. Initially Kaplan-Meier curves (univariate analysis) for time to culture conversion and time to reversion were explored to test the proportional hazard (PH) assumption across independent variable categories. The assumption of proportional hazards was also verified using Schoenfeld residuals from the Cox models [11]. The estimated hazard ratios (HRs) and 95% confidence intervals are presented. Variables were first included in a bivariate Cox model and those with a p-value <0.10 were included in multivariate models. Time dependent hazard ratios were plotted for independent variables that violated the PH assumption.

To interpret time to culture conversion in bivariate and multivariate regression models HR<1 indicates lower chance of and longer time to culture conversion (positive event/outcome) with respect to reference category of independent variable. To interpret time to reversion after culture conversion in logistic regression model, HR>1 indicates higher hazard of reversion (negative event/outcome) with respect to reference category of independent variable. The hazard ratios were also complemented with median time to event.

Logistic and multinomial logistic regression models were used to determine key factors significantly associated with poor treatment outcomes. For logistic regression, outcomes of death, loss to follow up, and treatment failure were combined as a single unfavorable outcome. For the multinomial logistic regression model, four categories were created with favorable outcomes (cured and treatment completed) as the reference category, and then separate outcomes for death, lost to follow up (LTFU) or treatment failure. The adjusted odds ratios (AOR) of each unfavorable outcome is presented with reference to favorable outcome (cured/treatment completed). An AOR>1 indicates higher odds of unfavorable outcome.

A subgroup analysis to test for association of SLDST results with treatment outcomes as dependent variable was performed using logistic regression model. All estimates were tested at 5% level of statistical significance (p value ≤ 0.05). We adhered to STROBE guidelines for reporting observational studies (S1 Checklist) [12].

### Ethical consideration

The protocol was reviewed and approved by the Central TB Division, Ministry of Health and Family Welfare, Government of India. This study used the PMDT records maintained as per the programme guidelines to collect the data on the study variables. Since the study collected the data from an established practice as per the programme guidelines, within the framework of routine care, individual patient consent and ethical approval was deemed unnecessary by the Central TB Division. However, the whole process of data management was done with shared confidentiality within the health staff of the participating treatment centers and the authors. No external funding was used for this analysis.

## Results

We describe a cohort of 3741 MDR-TB patients (including RR-TB) from 15 DR-TB Centers from seven states of India, among whom 29 (0.8%) are excluded due to inadequate data, yielding a cohort of 3712 patients for analysis (S1 Fig). Of these, 2735 (73.6%) had culture conversion at a median time of 100 days (IQR 92–125), among whom 506 (18.5%) had culture reversion at a median time of 279 days (IQR 202–381). Among 3712 MDR-TB patients, final definite treatment outcomes were available in 2264 (60.9%) MDR-TB patients registered on treatment while the remaining 1448 (39.0%) patients did not have a final treatment outcome, part of who were expected to have successful outcomes, as they were reported to be either still on treatment under 24 months (871, 23.5%) or their treatment outcomes were yet to be recorded (577, 15.5%) at the time of data collection. Only 781 (34.5%) of 2264 MDR-TB patients were reported to have favorable treatment outcomes (cured/treatment completed), while 1483 (66%) had unfavorable treatment outcomes. Also 664 (29.3%) had died and 670 (29.6%) were LTFU.

The characteristics of the 3712 MDR-TB patients are detailed in Table 1. 2564 (69.1%) patients were males and a majority 2679 (72.2%) were young (15–44 years) with a median age of 35 years (IQR 25, 45). Only 58 (1.6%) were co-infected with HIV and 293 (7.9%) had diabetes. Nearly half of them had an initial registration type as treatment after failure (1892, 51.0%) while more than a quarter had relapse (1003, 27.0%). Exposed to a median treatment duration of 11 months (IQR 7, 16); 3026 (81.5%) patients had prior exposure to re-treatment regimen taken twice (erstwhile Category II) and 3011 (81.1%) had most recent treatment from government source. With a median weight of 42 kg at baseline (IQR 36, 49), more than half of the patients were undernourished with baseline BMI <18 (1957, 52.7%) and almost half had baseline cavitation on chest X-ray (1698, 45.7%). The diagnosis of MDR-TB was established in 3042 (82.0%) patients with phenotypic method and in 670 (18.0%) with FL-LPA. The median months of delay in treatment initiation after diagnosis of MDR-TB was very high at 4.23 months (IQR 3.4, 5.7) with phenotypic methods, but was also high at 1.2 (IQR 0.7, 1.8) months with LPA. In 2407 (64.8%) of MDR-TB patients with H and R resistance, additional resistance was also observed to either E (1710, 46.1%) and/or S (2259, 60.8%). In a subgroup of 104 MDR-TB patients with additional baseline SLDST results available, Ofx resistance was present in 62 (59.6%). Nearly half of the patients had experienced any weight gain of 1 kg or more at 6 months (1834, 49.4%) and 12 months (1737, 46.8%) of treatment. Adherence to MDR-TB treatment was provided under daily observation by a health care provider or trained community volunteer. It was observed that only 849 (22.9%) and 649 (17.4%) patients missed ≥ 7doses in the intensive (IP) and continuation phase (CP) respectively.

**Table 1:** Characteristics of MDR-TB cohort.

Variable	N	n (%)	Median (IQR)
**Gender**	3712		
Female		1148 (30.9)	
Male		**2564 (69.1)**	
**Age (years)**	3712		35.0 (25, 45)
**Age group (years)**	3712		
<15	1	52 (1.4)	
15–44	0.62(02)	**2679 (72.2)**	
45–64	0.56(75)	881 (23.7)	
>64	0.56(01)	100 (2.7)	
**HIV status**	3712		
Negative		3597 (96.9)	
Positive		**58 (1.6)**	
Unknown		57 (1.5)	
**Diabetes**	3712		
No		3419 (92.1)	
Yes		**293 (7.9)**	
**Initial registration type**	3712		
Relapse		**1003 (27.0)**	
Treatment after LTFU	0.65 (0	527 (14.2)	
Treatment after Failure	0.94.02)	**1892 (51.0)**	
New (contacts)	0. 0.96)	175 (4.7)	
Others (other than above types)	0.88(10)	115 (3.1)	
**Number of previous treatment episodes**	3337		2 (1, 3)
**Re-treatment regimen taken twice**	3702		
No		676 (18.3)	
Yes		**3026 (81.7)**	
**Duration of previous treatment episodes (months)**	3319		11 (7, 16)
**Source of most recent previous treatment**	3712		
Government		**3011 (81.1)**	
Private		267 (7.2)	
Other^#^		434 (11.7)	
**Body mass index**	3712		
<18		**1957 (52.7)**	
≥18		1755 (47.3)	
**Baseline weight in Kg.**	3712		42 (36, 49)
**Cavitation**	3712		
No		1536 (41.3)	
Yes		**1698 (45.9)**	
Unknown		478 (12.9)	
Drug Susceptibility Testing (DST) method*	3712		
Phenotypic		**3042 (82.0)**	
Genotypic		670 (18.0)	
**Treatment delay by DST method (months)**	3691		
Phenotypic		3027 (82.0)	**4.23 (3.4, 5.7)**
Genotypic		664 (18.0)	1.17 (0.7, 1.8)
First line DST* **(L J)**	3712		
**Ethambutol (E)**			
Resistance		**1710 (46.1)**	
Susceptible		1307 (35.2)	
Unknown		695 (18.7)	
**Streptomycin (S)**			
Resistance		**2259 (60.9)**	
Susceptible		766 (20.6)	
Unknown		687 (18.5)	
First line DST* **(L J or LPA)**	3702		
R only		187 (5.0)	
HR only		1058 (28.5)	
HR combination		**2407 (64.8)**	
R combination		50 (1.4)	
Second line DST* (Gujarat subgroup)	104		
Any Ofloxacin (Ofx) resistance		**62 (59.6)**	
Kanamycin (Km) resistance		8 (7.7)	
Ethionamide (Eto) resistance		28 (26.9)	
XDR-TB		6 (5.8)	
**Weight change (6 months)**	3712		
No change		537 (14.5)	
Any Loss	1	480 (12.9)	
Any Gain	1.938)	**1834 (49.4)**	
Not available	2, 2.43)	861 (23.2)	
**Weight change (12 months)**	3712		
No change		276 (7.4)	
Any Loss		377 (10.2)	
Any Gain		**1737 (46.8)**	
Not available	1.9.27)	1322 (35.6)	
**Treatment adherence (Intensive Phase)**	3712		
<7 missed doses		2718 (73.2)	
≥7 missed doses		**849 (22.9)**	
Not available		145 (3.9)	
**Treatment adherence (Continuation Phase)**	3712		
<7 missed doses		2627 (70.8)	
≥7 missed doses		**649 (17.4)**	
Not available		436 (11.8)	

# Other sector health facilities like central government health schemes, railways, mines, defense services, employee states insurance scheme etc.

*Results from pre-treatment specimens only.

Initial culture conversion was observed in 2735 (73.7%) patients (S1 Fig). Multivariate analysis for time to culture conversion (Table 2) adjusted for age, gender and HIV status revealed several risk factors that significantly (p value ≤ 0.05) reduced the chances of culture conversion and prolonged the median time to conversion. Type of patients registered as treatment after LTFU (HR 0.68, p value <0.001) or treatment after failure (HR 0.86, p value 0.004) had significantly lower chances of culture conversion and had longer median time to culture conversion with reference to the type Relapse. Patients with baseline BMI <18 (HR 0.73, p value <0.001) and patients with chest X-ray showing cavitation (HR 0.84, p value <0.001) also had significantly lower culture conversion.

**Table 2:** Bivariate and multivariate survival models for time to culture conversion. (Note: HR value <1 means lower chances of and longer time to culture conversion (positive event/outcome).

Variable	Bivariate			Multivariate (N = 2735)*		Median time to culture conversion
	N	HR	(95% CI); p-value	HR	(95% CI); p-value	
Gender	3712					
Female (Ref)		1		1		107(104,112)
Male		0.95	(0.88,1.03);0.23	0.98	(0.90,1.08);0.728	109(106,112)
Age group	3712					
<15		1.42	(1.06,1.89);0.02	1.09	(0.73,1.65);0.669	98(95,105)
15–44 (Ref)		1		1		108(106,112)
45–64		0.95	(0.87,1.04);0.28	0.94	(0.96,1.192);0.24	113(106,120)
>64		1.21	(0.95,1.54);0.12	1.08	(0.93,1.62);0.15	101(97,111)
HIV status^†^	3655					
Negative (Ref)		1				108(111,)
Positive		1.02	(0.75,1.37);0.921			111(151,112)
Diabetes^†^	3712					
No (Ref)		1		1		109(106,112)
Yes		1.26	(1.10,1.44);0.001	1.16	(0.99,1.35);0.052	104(101,110)
**Initial registration type**	3712					
Relapse (Ref)		1		1		103(100,106)
LTFU		0.65	(0.58,0.74);<0.001	**0.68**	**(0.58,0.78); < .001**	**131(125,142)**
Treatment after Failure		0.94	(0.86,1.03);0.167	**0.86**	**(0.78,0.95);0.004**	**107(105,111)**
New contacts		0.82	(0.68,0.98);0.035	0.82	(0.66,1.00);0.053	120(104,140)
Others		0.87	(0.69,1.09);0.213	0.79	(0.61,1.02);0.074	105(98,120)
**Previous treatment episodes**						
Number of episodes	3337	0.89	(0.85,0.93); 0.001	0.95	(0.89,1.02);0.143	
Re-treatment regimen taken twice	3702					
No (Ref)		1		1		107(105,109)
Yes		0.77	(0.69,0.86); 0.001	1.04	(0.91,1.19);0.592	119(110,125)
Duration (months)	3319	0.99	(0.98,0.99);0.001	0.99	(0.98,1.01);0.517	
Source of most recent previous treatment	3712					
Government (Ref)		1		1		109(106,112)
Private		0.75	(0.64,0.88);<0.001	0.88	(0.74,1.04);0.127	127(117,142)
Other^#^		1.27	(1.13,1.42);<0.001	0.82	(0.59,1.13);0.22	99(97,102)
**Body mass index**	3712					
≥18 (Ref)		1		1		102(101,105)
<18		0.70	(0.65,0.76);<0.001	**0.73**	**(0.67,0.81);<0.001**	**118(114,122)**
**Cavitation**^†^	3234					
No (Ref)		1		1		105(103,108)
Yes		0.81	(0.75,0.87);<0.001	**0.84**	**(0.77,0.91);<0.001**	**115(110,119)**
Treatment delay by DST method	3701					
Phenotypic (Ref)		1		1		106(104,108)
Genotypic		0.71	(0.64,0.79);<0.001	0.65	(0.36,1.16);0.141	125(118,131)
First line DST (L J)						
E—L J^†^	3017					
Resistance (Ref)		1		1		107(104,111)
Susceptible		1.08	(0.99,1.17);0.07	0.65	(0.36,1.15);0.141	104(102,108)
S—L J^†^	3025					
Resistance (Ref)		1		1		106(104,110)
Susceptible		1.10	(0.99,1.20);0.052	1.06	(0.88,1.26);0.552	105(102,109)
First line DST (LPA)	3702					
R only (Ref)		1		1		124(108,137)
HR only		1.20	(0.99,1.45);0.054	1.00	(0.82,1.22);0.998	111(106,118)
HR combination		1.33	(1.11,1.59);0.002	0.98	(0.73,1.31);0.894	106(104,109)
R combination		1.25	(0.87,1.79);0.235	1.03	(0.67,1.59);0.89	111(96,127)
Weight change (6 m)^†^	2851					
No change (Ref)		1		1		110(103,120)
Any Loss		1.01	(0.87,1.16);0.896	1.01	(0.87,1.19);0.824	108(104,115)
Any Gain		1.18	(1.06,1.31);0.003	1.03	(0.91,1.16);0.614	104(102,106)
Weight change (12m)^†^	2890					
No change (Ref)		1		1		102(99,108)
Any Loss		0.85	(0.71,1.00);0.06	0.83	(0.69,1.01);0.057	111(104,121)
Any Gain		1.20	(1.05,1.38);0.009	1.12	(0.97,1.31);0.133	102(101,104)
**Treatment adherence (IP)**^†^	3567					
<7 missed doses (Ref)		1				102(104,137)
≥7 missed doses		**0.47**	**(0.42,0.52);<0.001**			**160(182,118)**

# Other sector health facilities like central government health schemes, railways, mines, defense services, employee states insurance scheme etc.

*Model is adjusted for age group and gender and HIV status is dropped from the analysis.

^**†**^ Estimates for the category “unknown” are not reported here

One variable of interest was treatment adherence with ≥7 missed doses during IP. We explored cut-off values of 6 to 30 for missed doses in IP using regression discontinuity analysis to assess the implication of choosing alternate cut-off dates [10] and found that the optimal cut-off value was 7 missed doses. Seven or more doses represent missing just <4% of the average 180 daily doses in IP. The main effect on culture positivity did not change for higher cut-off values. The density curves for missed doses at IP and CP in regression discontinuity analysis also showed a marked change at the cut-off of 7 missed doses. In logistic regression analysis, ≥7 missed doses during IP was significantly associated with culture conversion (Table 2). We were unable to include this variable in the multivariate Cox proportional hazard model for culture conversion, as it violated the proportional hazards assumption (S2 Fig).

Bivariate regression analysis for culture reversion (Table 3) observed in 506 patients out of those who had initial culture conversion revealed several risk factors that significantly enhanced the hazard of culture reversion at p value ≤ 0.05. These include patients exposed to re-treatment regimen taken twice (HR 1.39, p value 0.02); who had any weight loss of 1 kg or more at 6 months of MDR-TB treatment (HR 1.58, p value 0.041); low adherence to treatment i.e. patients with ≥ 7 missed doses in IP (HR 1.66, p value <0.001) and CP (HR 1.38, p value 0.017) and patients with longer time to culture conversion (HR 2, p value 0.001).

**Table 3:** Factors significantly associated with culture reversion with bivariate regression model. (Note: HR value >1 means higher hazard of reversion after culture conversion (negative event/outcome).

Variable	Estimates (N = 506)		Median time to reversion after culture conversion
	HR	(95% CI); p-value	
Gender			
Female (Ref)	1		172(115,166)
Male	0.94	(0.74,1.21);0.651	331(128,176)
Age group			
<15 (Ref)	1		11(60,287)
15–44	0.79	(0.35,1.79);0.585	362(122,161)
45–64	0.97	(0.42,2.23);0.946	117(126,195)
>64	0.98	(0.34,2.83);0.974	13(90,183)
HIV status^†^			
Negative (Ref)	1		488(126,159)
Positive	1.71	(0.84,3.44);0.137	10(60,327)
Diabetes			
No (Ref)	1		144(97,217)
Yes	0.93	(0.62,1.42);0.749	146(128,166)
Initial registration type			
Relapse (Ref)	1		149(117,185)
LTFU	1.10	(0.74,1.63);0.642	156(106,182)
Treatment after failure	1.12	(0.85,1.47);0.406	144(123,168)
New contacts	1.08	(0.61,1.90);0.801	196(63,297)
Others	0.49	(0.18,1.33);0.159	89(64,136)
Previous treatment episodes			
Number of previous episodes	1.10	(0.97,1.24);0.145	
**Re-treatment regimen taken twice**			
No (Ref)	1		151(126,181)
Yes	**1.39**	**(1.05,1.85);0.02**	**144(123,166)**
Duration of previous episodes (months)	1.01	(0.99,1.03);0.082	
Source of most recent previous treatment			
Government (Ref)	1		146(128,166)
Private	1.08	(0.69,1.71);0.731	115(94,185)
Other^#^	0.98	(0.69,1.42);0.953	166(116,186)
Body mass index			
≥18 (Ref)	1		148(126,170)
<18	1.20	(0.95,1.51);0.117	144(117,166)
Cavitation^†^			
No (Ref)	1		153(126,174)
Yes	1.11	(0.87,1.42);0.388	141(122,172)
Treatment delay by DST method			
Phenotypic (Ref)	1		144(124,165)
Genotypic	0.83	(0.59,1.16);0.275	151(123,188)
First line DST (L J)			
E–L J^†^			
Resistance (Ref)	1		147(122,169)
Susceptible	0.96	(0.75,1.24);0.757	136(116,176)
S–L J^†^			
Resistance (Ref)	1		141(120,159)
Susceptible	1.06	(0.80,1.40);0.673	157(121,204)
First line DST (LPA)			
R only (Ref)	1		166(105,240)
HR only	0.88	(0.56,1.38);0.581	157(134,197)
HR combination	1.13	(0.73,1.74);0.579	135(119,158)
R combination	1.07	(0.36,3.11);0.898	68(61,76)
**Weight change (6 months)**^†^			
No change (Ref)	1		141(106,187)
Any Loss	**1.58**	**(1.02,2.45);0.041**	**165(120,204)**
Any Gain	1.41	(0.98,2.04);0.065	146(126,166)
Weight change (12 months)^†^			
No change (Ref)	1		134(105,186)
Any Loss	1.30	(0.79,2.14);0.296	151(129,170)
Any Gain	0.96	(0.63,1.47);0.859	176(118,208)
**Treatment adherence (IP)**^†^			
<7 missed doses (Ref)	1		196(63,359)
≥7 missed doses	**1.66**	**(1.26,2.19);<0.001**	**135(105,181)**
**Treatment adherence (CP)**^†^			
<7 missed doses (Ref)	1		140(123,163)
≥7 missed doses	**1.38**	**(1.06,1.81);0.017**	**159(124,188)**
**Time to culture conversion**	**2.00**	**(1.84,2.04);0.001**	

# Other sector health facilities like central government health schemes, railways, mines, defense services, employee states insurance scheme etc.

†Estimates for the category “unknown” are not reported here.

In the treatment outcome analysis, 871 patients who were still on treatment for less than 24 months and 577 patients for whom outcomes were unavailable at the time of data collection were excluded from the model (S1 Fig). Median survival time for patients with low BMI (15.5 months) was less than that for normal BMI (18.8 months) (S3 Fig). Similarly, the median survival for patients who missed less than 7 doses in IP was significantly higher (23.0 months) than those who missed 7 doses or more (11.5 months).

Factors significantly associated with the combined ‘unfavorable’ treatment outcomes (i.e. LTFU, treatment failure, died) in 2264 patients using logistic regression model (Table 4) include male gender (OR 1.38, p value 0.01); BMI <18 (OR 1.64, p value <0.001); low adherence to treatment i.e. patients with ≥ 7 missed doses in IP (OR 2.76, p value <0.001) and in CP (OR 1.51, p value <0.001); and greater number (OR 1.29, p value <0.001) of previous treatment episodes. Susceptibility to E was observed to be protective against unfavorable treatment outcomes (OR 0.65, p value 0.01).

**Table 4:** Factors significantly associated with unfavorable treatment outcomes from logistic regression analysis. (Note: OR>1 indicates higher odds of unfavorable outcome).

Variable	Odds ratio	N = 2264; (95% CI); p = value
**Gender**		
Female (Ref)	1	
Male	**1.38**	**(1.08,1.76);0.01**
Age group		
<15 (Ref)	1	
15–44	1.07	(0.27,4.33);0.92
45–64	1.06	(0.26,4.36);0.94
>64	2.58	(0.53,12.65);0.24
HIV status^†^		
Negative (Ref)	1	
Positive	1.08	(0.5,2.34);0.84
Unknown	0.65	(0.26,1.59);0.35
Diabetes		
No (Ref)	1	
Yes	0.96	(0.63,1.44);0.83
Initial registration type		
Relapse (Ref)	1	
LTFU	1.26	(0.85,1.86);0.25
Treatment after failure	1.01	(0.77,1.33);0.93
New contacts	1.44	(0.69,3.02);0.34
Others	1.74	(0.85,3.57);0.13
Previous treatment episodes		
No. of previous episodes	1.29	(1.09,1.53);< 0.001
Retreatment regimen taken twice		
No (Ref)	1	
Yes	0.8	(0.56,1.15);0.23
Duration of previous episodes	0.98	(0.95,1);0.02
Source of most recent previous treatment		
Government (Ref)	1	
Private	0.89	(0.6,1.33);0.57
Other^#^	0.53	(0.25,1.14);0.1
**Body mass index**		
≥18 (Ref)	1	
**<18**	**1.64**	**(1.28,2.11);< 0.001**
Cavitation^†^		
No (Ref)	1	
Yes	1.1	(0.87,1.39);0.44
Treatment delay by DST method		
Phenotypic (Ref)	1	
Genotypic	1.06	(0.1,11.05);0.96
**E–L J**^†^		
Resistance (Ref)	1	
**Susceptible**	**0.65**	**(0.48,0.89);0.01**
S–L J^†^		
Resistance (Ref)	1	
Susceptible	0.63	(0.39,1.03);0.07
First-line drug resistance		
R only (Ref)	1	
HR only	1.67	(0.74,3.77);0.22
HR combination	0.73	(0.27,1.98);0.53
R combination	2.72	(0.57,12.96);0.21
Weight change at 6 months^†^		
No change (Ref)	1	
Any Loss	0.9	(0.59,1.37);0.63
Any Gain	1.05	(0.76,1.47);0.75
Weight change at 12 months^†^		
No change (Ref)		
Any Loss	1.38	(0.81,2.33);0.23
Any Gain	0.92	(0.59,1.43);0.7
**Treatment adherence (IP)** ^**†**^		
<7 missed doses (Ref)	1	
**≥7 missed doses**	**2.76**	**(2.03,3.77);< 0.001**
**Treatment adherence (CP)** ^**†**^		
<7 missed doses (Ref)	1	
**≥7 missed doses**	**1.51**	**(1.15,1.98);< 0.001**

# Other sector health facilities like central government health schemes, railways, mines, defense services, employee states insurance scheme etc.

†Estimates for the category “unknown” are not reported here.

Multinomial logistic regression model to determine key factors significantly associated with the outcomes of loss to follow-up (n = 670), treatment failure (n = 169), and death (n = 644) among the 2264 patients showed similar risk factors (Table 5) as with combined unfavorable treatment outcomes (Table 4). In addition, factors significantly associated with death included cavitation (AOR 1.41, p value 0.02); genotypic DST (AOR 1.41, p value 0.02) with reference to phenotypic DST method; previous registration type others (AOR 2.68, p value 0.02) with reference to relapse had increased odds for death while any weight gain at 12 months (AOR 0.52, p value 0.02) and susceptibility to S (AOR 0.45, p value 0.01) were observed to be protective against death i.e. predicted better survival.

**Table 5:** Factors significantly associated with poor outcomes as compared to cured/completed treatment from multinomial logistic regression analysis. (Note: AOR>1 indicates higher odds of LTFU or Treatment Failure or Died in respective columns).

Variable	LTFU (N = 670)		Treatment Failure (N = 169)		Died (N = 644)	
	AOR	(CI);p-value	AOR	(CI);p-value	AOR	(CI);p-value
**Gender**						
Female (Ref)	1		1		1	
Male	**1.91**	**(1.38,2.66);<0.001**	**1.6**	**(1.1,2.33);0.02**	1.01	(0.75,1.36);0.94
Age group						
<15 (Ref)	1		1		1	
15–44	2.3	(0.23,23.23);0.48	668896.6*	(0,.);0.99	0.68	(0.16,2.98);0.61
45–64	2.19	(0.21,22.46);0.51	548969*	(0,.);0.99	0.84	(0.19,3.78);0.82
>64	6.38	(0.53,76.79);0.14	1165306*	(0,.);0.99	2.29	(0.41,12.79);0.35
HIV status						
Negative (Ref)	1		1		1	
Positive	0.55	(0.17,1.77);0.32	1.82	(0.7,4.71);0.22	0.76	(0.26,2.26);0.63
Unknown	1.24	(0.42,3.69);0.7	0.38	(0.04,3.31);0.38	0.73	(0.22,2.37);0.6
Diabetes						
No (Ref)	1		1		1	
Yes	0.99	(0.56,1.73);0.97	1.31	(0.71,2.41);0.39	0.92	(0.53,1.61);0.78
**Initial registration type**						
Relapse (Ref)	1		1		1	
LTFU	1.32	(0.81,2.14);0.27	1.19	(0.66,2.13);0.57	1.33	(0.83,2.13);0.24
Treatment after failure	0.72	(0.5,1.05);0.09	0.95	(0.61,1.47);0.82	0.96	(0.67,1.36);0.82
New contacts	1.2	(0.49,2.95);0.69	1.96	(0.67,5.72);0.22	1.94	(0.82,4.56);0.13
Others	0.84	(0.31,2.3);0.74	1.62	(0.53,4.94);0.39	**2.68**	**(1.2,5.99);0.02**
Previous treatment episodes						
Number of previous episodes	0.96	(0.75,1.22);0.72	1.04	(0.78,1.39);0.78	1.18	(0.93,1.48);0.17
Duration of previous episodes (months)	1	(0.97,1.03);0.89	0.99	(0.96,1.03);0.75	0.99	(0.97,1.02);0.59
Re-treatment regimen taken twice						
No (Ref)	1		1		1	
Yes	1.02	(0.63,1.64);0.94	1.12	(0.64,1.93);0.7	1.04	(0.67,1.61);0.88
**Source of most recent previous treatment**						
Government (Ref)	1		1		1	
Private	1.07	(0.62,1.83);0.81	1.64	(0.9,2.99);0.1	0.86	(0.52,1.43);0.55
Other^#^	0.54	(0.19,1.56);0.26	**0.18**	**(0.04,0.86);0.03**	0.52	(0.19,1.42);0.2
**Body mass index**						
≥18 (Ref)	1		1		1	
<18	**1.6**	**(1.12,2.29);0.01**	**1.82**	**(1.2,2.76);0.01**	**4.89**	**(3.4,7.06);<0.001**
**Cavitation**						
No (Ref)	1		1		1	
Yes	0.98	(0.72,1.34);0.91	0.89	(0.62,1.26);0.5	**1.41**	**(1.05,1.91);0.02**
Unknown	1.72	(0.88,3.36);0.11	0.27	(0.06,1.23);0.09	1.53	(0.78,3.01);0.22
**Treatment delay by DST method**						
Phenotypic (Ref)	1		1		1	
Genotypic	1.04	(0.07,15.31);0.98	0.55	(0.02,13.63);0.72	**1.41**	**(1.05,1.91);0.02**
**First line DST (L J)**						
**E–L J**						
Resistance (Ref)	1		1		1	
Susceptible	**0.61**	**(0.41,0.91);0.02**	**0.6**	**(0.37,0.97);0.04**	1.53	(0.78,3.01);0.22
**S–L J**						
Resistance (Ref)	1		1		1	
Susceptible	0.6	(0.32,1.15);0.12	0.63	(0.31,1.29);0.21	**0.45**	**(0.24,0.84);0.01**
Unknown	2.41	(0.16,35.11);0.52	4.3	(0.17,107.94);0.38	0.6	(0.04,9.84);0.72
First line DST (LPA)						
R only (Ref)	1		1		1	
HR only	2.19	(0.86,5.57);0.1	1.57	(0.46,5.35);0.48	1.48	(0.61,3.59);0.39
HR combination	0.72	(0.22,2.41);0.6	0.72	(0.16,3.28);0.67	0.53	(0.17,1.71);0.29
R combination	4.88	(0.82,29.03);0.08	3.46*	(0,);0.98	3.62	(0.64,20.55);0.15
Weight change (6 months)						
No change (Ref)	1		1		1	
Any Loss	0.63	(0.36,1.11);0.11	1.99	(1.04,3.79);0.04	0.81	(0.49,1.35);0.41
Any Gain	0.95	(0.62,1.47);0.83	1.59	(0.92,2.74);0.1	0.92	(0.62,1.37);0.68
**Weight change (12 months)**						
No change (Ref)	1		1		1	
Any Loss	2.27	(0.97,5.34);0.06	1.12	(0.52,2.41);0.77	1.34	(0.7,2.55);0.38
Any Gain	1.7	(0.81,3.57);0.16	1.08	(0.56,2.09);0.81	**0.52**	**(0.3,0.9);0.02**
**Treatment adherence (IP)**						
<7 missed doses (Ref)	1		1		1	
**≥7 missed doses**	**4.36**	**(2.97,6.39); <0.001**	**2.29**	**(1.46,3.59);** **<0.001**	**2.13**	**(1.46,3.12);** **<0.001**
Unknown	0.05	(0.01,0.16); <0.001	0.14	(0.01,1.8); 0.13	0.08	(0.02,0.27); <0.001
**Treatment adherence (CP)**						
<7 missed doses (Ref)	1		1		1	
**≥7 missed doses**	**1.85**	**(1.27,2.7); <0.001**	**1.9**	**(1.29,2.79);** **<0.001**	0.98	(0.67,1.42); 0.91
Unknown	36.69	(13.7,98.4); <0.001	1.89	(0.37,9.64);0.44	22.06	(8.22,59.21); <0.001

# Other sector health facilities like central government health schemes, railways, mines, defense services, employee states insurance scheme etc.

* undefined due to very low cell frequencies in the cured/completed treatment category.

Of the subgroup of 104 MDR-TB patients (treatment failures of first line regimen) with additional baseline SLDST results available, 62 patients had Ofx resistance. Treatment outcomes in this subgroup stratified by DST to Ofx (Table 6) revealed low cure rates (25.8%), high mortality (27.4%), LTFU (22.6%) and treatment failure (21.0%) rates in MDR-TB patients with additional Ofx resistance as compared to 54.8%, 23.8%, 11.9% and 7.1% in those with Ofx susceptibility.

**Table 6:** Subgroup analysis of treatment outcome on MDR- TB patients with baseline SL DST (N = 104).

Treatment Outcomes	Ofloxacin Susceptible		Ofloxacin Resistant	
	N	%	N	%
Cured	23	54.8%	**16**	**25.8%**
Treatment Completed	0	0.0%	**2**	**3.2%**
Treatment Failure	3	7.1%	**13**	**21.0%**
Lost to Follow Up	5	11.9%	**14**	**22.6%**
Died	10	23.8%	**17**	**27.4%**
Treatment stopped	0	0.0%	**1**	**1.6%**
Total	42	100.0%	62	100.0%

In Table 7, we studied baseline SLDST and its association with time to culture conversion and time to culture reversion using separate cox regression models. For the same factors we used logistic regression analysis for treatment outcomes dichotomized as favorable or unfavorable. We found that susceptibility to Ofx (HR 1.97, p value 0.006) and Km (HR 4.28, p value 0.043) has significantly doubled and quadrupled chances for culture conversion (p value ≤ 0.05) compared to baseline resistance. Moreover, baseline susceptibility to Ofx (AOR 0.34, p value 0.014) has also significantly reduced odds of unfavorable treatment outcomes (p value ≤ 0.05) compared to baseline resistance.

**Table 7:** Baseline second line DST results as factors significantly associated with time to culture conversion, time to culture reversion and unfavorable treatment outcomes using survival and logistic regression analysis. (Note: HR>1 indicates higher chances of culture conversion, higher hazard of reversion after conversion and higher hazard of unfavorable treatment outcome in the respective column).

Baseline Second line DST Variable	Time to culture conversion (n = 104)	Time to culture reversion (n = 66)	Unfavorable Treatment outcome (n = 104)
	**HR (95% CI);p-value**	**HR (95% CI);p-value**	**AOR (95% CI);p-value**
**Ofloxacin**			
Resistance (Ref)	1	1	1
Susceptible	**1.97 (1.21,3.20);0.006**	0.37 (0.11,1.17);0.089	**0.34 (0.15,0.78);0.01**
**Kanamycin**			
Resistance (Ref)	1	1	1
Susceptible	**4.28 (1.05,17.5);0.043**	NA	0.53 (0.10,2.74);0.446
Ethionamide			
Resistance (Ref)	1	1	1
Susceptible	1.47 (0.83,2.62);0.188	0.54 (0.18,1.62);0.273	0.45 (0.17,1.19);0.11

## Discussion

MDR-TB patients in the initial cohorts diagnosed and treated under the RNTCP PMDT services in India experienced low culture conversion, high culture reversion, and unacceptably poor treatment outcomes. In context, MDR-TB treatment is difficult. Only 52% of MDR-TB patients initiated on treatment in the 2013 cohort were successfully treated globally [1] India has consistently reported low treatment success rates (46%) of MDR-TB patients managed under the national programme in the past few years. [1,13] These very concerning results generated considerable introspection. One possible consideration was that the initial cohorts in India were a particularly difficult set of patients to treat, with prolonged treatment history, chronic disease and delayed MDR-TB diagnosis. Most of them were heavily treatment experienced with four in five patients exposed to re-treatment regimen taken twice. These patients were offered DST very late in the disease course when on TB treatment. Four in five patients were offered phenotypic DST (solid LJ media) that takes 4–5 months to return a laboratory confirmation of MDR-TB. By the time they were diagnosed with MDR-TB, more than half of the patients in the cohort had cavitation in the lungs and a very low body-mass index. Regardless of these severities of disease in the cohort, the standard MDR-TB regimen under RNTCP performed poorly in rendering these patients noninfectious. A quarter of patients never achieved culture conversion; even among those who converted, one in five reverted back to culture-positive.

Common factors significantly associated with lower culture conversion included baseline undernutrition (BMI <18) [14,15], baseline cavitation [15] and any weight loss during treatment. Additionally, males [16], nonadherence (≥ 7 missed doses in IP and CP)[14], and patients with prior treatment exposure to multiple regimens and for longer duration [15,16] had greater risk of unfavorable treatment outcomes. Subgroup analysis in the limited number of patients with SLDST found Ofx or Km resistance strongly associated with delayed culture conversion, and Ofx susceptibility protective against unfavorable treatment outcomes [15,16]. The association between treatment adherence as a dichotomous variable, effectively splitting those patients who were fully adherent (i.e. missing just <4% of IP doses), limited our ability to comment on the dose effect of nonadherence. Our data were not structured to allow more detailed analysis of time-based adherence. Moreover, there are limitations on reported adherence data by health workers who may be reluctant to document actual nonadherence. Regardless, the striking association between *any* nonadherence and poor culture conversion and poor outcomes strongly indicates that the association between adherence and treatment success needs detailed and dedicated exploration in future studies. This is further supported by Podewils LJ et.al. who reported patients who missed more than 10% of treatment doses in the first 6 months (~18 doses) had a significantly higher risk of failing to convert to a negative culture at the 6 month, 6–12 months and 12–18 months follow-up as well as BMI <18 to be significantly associated with poor treatment outcomes in Phillipines [14].

The study design has a limitation that it is restricted to the variables that had to be extracted from the routine PMDT programme records. Thus, we could not capture data on potentially important factors observed significantly associated with unfavorable treatment outcomes in some other studies [17,18,19,20] such as time-dependent adherence, adverse events, psychosocial needs, economic situation of the patients, migrant population, any catastrophic cost other than the diagnosis and drugs for MDR-TB provided free under the RNTCP services etc. Such data were not routinely recorded. The inter-reader variation in the grades of chest X-ray classification [7] as mild, moderately advanced and far advanced, was high during data validation and hence only cavitary or non-cavitary readings were included in the analysis. Treatment outcomes were not available for 15.5% of the patients as data collection was closed in June 2012. However, the basic characteristics of the patient groups with known and unknown treatment outcomes showed no meaningful differences. Only a small subgroup of patients had SLDST data, limited to the consecutive cohort of patients from one state, Gujarat, in the earliest days of PMDT program implementation. This subgroup of patients was heavily treatment experienced and may not be representative of the larger nationally-sourced patient population in the rest of the analysis. For most patients, SLDST results were simply not available due to the lack of laboratory capacity for SLDST during the study period. The association of second line drug susceptibility and improved outcomes, accordingly, should be interpreted judiciously. Further, as there was no policy of monitoring long term follow up of successfully treated MDR-TB patients under RNTCP, we also could not perform relapse surveillance which could have affected the results.

What are the implications of these findings? The standard MDR-TB regimen under RNTCP may be inadequate in a surprising proportion of patients. Although we had limited data on second-line anti-TB drug resistance, in the subgroup of patients with baseline second-line DST results, Ofx resistance at baseline [15,16] halved the cure rates and tripled the probability of treatment failure in patients treated with standard MDR-TB regimen. Ofx resistance has also been found to be very common among MDR-TB isolates in India, in the range of 28–50% in various published studies and from programme surveillance data from RNTCP [21,22,23,24,25]. Although discouraged by RNTCP and regulated under Schedule H1 of Drug and Cosmetics Rules (4^th^ amendment) 2013 [26], fluoroquinolones (FQ) are commonly used as first-line drugs in patients with respiratory diseases by the practitioners across India [27]. Our study also revealed protective factors like baseline susceptibility to E, S, Ofx, Km and any weight gain at 12 months (indicating effectiveness of treatment) to be significantly associated with better conversion, favorable outcomes and better survival. These collectively emphasizes the fact that a national policy of universal DST for early diagnosis and baseline DST to all first and second line drugs for whom standardized DST methods are available [28] and endorsed by WHO was necessary. WHO endorsed rapid molecular tests like Xpert MTB/Rif and LPA (FL, SL) can now be intelligently used for rapid testing of presumptive patients to triage them for presence of RR-TB, RS-TB with H mono resistance on FL-LPA and RR-TB with FQ and/or SLI resistance on SL-LPA. Such a rapid triage of patients can allow careful approach to regimen design guided by the baseline rapid molecular test results (H, R, FQ, and SLI) and is a necessary intervention for improving treatment outcomes in India. Recently, the interim results of the STREAM Stage 1 trial showed that the shorter MDR-TB regimen had around 80% efficacy, similar to WHO recommended longer regimen, among FQ and SLI susceptible patients with clear advantage in reduction of pill burden and cost to the programmes [29]. Augmented MDR-TB regimens with newer drugs like Bedaquiline, Delamanid, Pretomanid offer more hope for higher treatment success rates particularly in MDR/RR-TB patients with baseline FQ and/or SLI resistance as evidence continues to emerge globally. The recent WHO-recommended shorter MDR-TB regimen offered to patients after ruling out baseline FQ and SLI resistance with SL-LPA and newer drug containing individualized regimen for patients with baseline FQ and/or SLI resistance on SL-LPA have promise with emerging evidence of substantially improving culture conversion and treatment outcomes among MDR/RR-TB patients [1,30,31,32]. Thus, we expect a more systematic algorithmic approach to DST guided regimens has the potential to improve treatment outcomes and survival of the MDR/RR-TB patients in India [25,33].

The other important risk factors that were strikingly and consistently associated with all unfavorable interim and final outcomes were poor treatment adherence i.e. ≥ 7 missed doses in IP & CP and undernutrition i.e. BMI <18. One major problem with MDR-TB therapy was toxicity warranting active drug safety monitoring and management mechanisms as this can often lead to missed doses or treatment interruption [20]. The interplay of toxicity and adherence requires further evaluation. Few prior studies have explicitly outlined the relationship between nonadherence and culture conversion or adverse outcomes. These findings highlight the low therapeutic index of the standard MDR-TB regimen, and also strengthen the case for counseling patients and family members as well as use of digital tools in monitoring treatment adherence enabling health workers to prioritize patients for prompt retrieval and support activities to even a few missed doses [15,34,35]. Undernutrition has been frequently associated with poor culture conversion and treatment outcomes in various studies [15,36,37,38,39]. This is substantiated by our finding and emphasizes the need for policies and enabling systems for a thorough nutritional assessment and necessary supplementation for MDR-TB patients [40]) and their family contacts to address undernutrition.

Unexpectedly, the patients diagnosed by genotypic DST (LPA) had a higher risk of death; we hypothesize that this may be due to survival bias in the patients diagnosed by phenotypic DST. Only those patients who started treatment were enrolled in this study, and as shown by a cascade of care analysis of MDR-TB patients from India [41], substantial patient loss occurs between identification and treatment initiation, which may be exacerbated with long turnaround time to results, as with phenotypic DST. Conversely, with genotypic DST, critically ill patients would have been likely enrolled in the treatment cohort and may have contributed to the excess fatality observed. We did not capture data from any MDR-TB patients who may have been lost before treatment initiation, and this finding thus merits separate analysis.

This is the first analysis to look at factors significantly associated with culture reversion among MDR-TB patients treated under RNTCP PMDT services in India. Reversion was common in this cohort, occurring among 506 (19%) of the 2735 patients who experienced initial culture conversion. Time to culture conversion, missing ≥7 days in intensive phase, missing ≥7 days in continuation phase and weight loss were each independently associated with reversion.

The WHO End TB Strategy encourages countries to accelerate towards universal DST; use of electronic/mobile health solutions for better systems of TB/DR-TB surveillance, faster transmission of results from rapid molecular tests as well as adherence monitoring; addressing nutritional assessment and supplementation as well as patient support systems to alleviate catastrophic expenditure and the impoverishing impact of MDR-TB on the patient and their family [1,35,42,43,44].

Early findings from this study informed further periodic analysis of programmatic data and global updates and facilitated national policy refinements on i) earlier MDR-TB diagnosis by improving access to rapid molecular drug susceptibility test (DST) (LPA and Xpert MTB-Rif) to a wider group of patients earlier during their disease course preferably at diagnosis of TB among previously treated patients and among new TB patients with higher vulnerability like PLHIV, children, contacts of MDR-TB; ii) baseline second line drug susceptibility test (SLDST) to enable early modification of standard MDR-TB regimen based on FQ and/or SLI resistance; iii) a DST guided approach to regimen design for the spectrum of DR-TB patterns; and iv) introducing counseling services at DR-TB centers across India. It also simultaneously facilitated programmatic improvements on i) rapid systematic scale-up of laboratory and PMDT services with nationwide coverage in 2013; ii) enhanced monitoring and evaluation through a policy on dedicated zonal and state performance reviews; iii) collaboration of RNTCP with pharmacovigilance programme of India for improving adverse drug reaction management and monitoring and iv) development of online case based patient tracking cum data management system (NIKSHAY) with information communication technology (ICT) based systems. These are expected to improve outcomes by promoting nationwide access to early rapid molecular diagnosis of MDR-TB before patients advance to cavitary disease or get exposed to multiple episodes and longer duration of TB treatment; prevent augmentation of further resistance through appropriate DST guided regimen designs; and promote weight gain of patients and their families through nutritional assessment and support as well as promote treatment adherence.

More recently, national guidelines for TB and PMDT were updated to align with the WHO End TB Strategy, updated WHO PMDT guidelines (2016) and other recent evidence based global updates. [45,46,47]. The factors associated with interim and final outcomes of MDR-TB revealed in our study are likely to be more rigorously addressed henceforth with these new RNTCP policy and plans for introduction and scale up of i) an algorithmic approach with universal DST to all diagnosed TB patients using Xpert MTB/Rif with baseline SLDST using FL/SL LPA to triage patients by DST patterns into H, R, FQ & SLI resistance; ii) DST guided treatment addressing the complete range of DR TB with tailored regimen for H mono/poly DR-TB, shorter MDR-TB regimen for MDR/RR-TB susceptible to FQ and SLI, Bedaquiline and other newer drugs containing regimen for MDR/RR-TB with FQ and/or SLI resistance; iii) active drug safety monitoring and management system; iv) social protection mechanism including nutritional assessment and support; v) ICT based patient tracking and adherence monitoring mechanisms and vi) promoting research and innovations. These are in line with the five priority actions, declared by WHO, needed to combat the MDR-TB epidemic from prevention to control [44,48].

## Conclusion

India’s initial MDR-TB cohorts treated under RNTCP experienced poor culture conversion, high level of culture reversion among those who were initially culture converted, and poor treatment outcomes, no better than the already low global average. To address the factors associated with poor treatment outcomes revealed in our study, a systematic multi-pronged approach would be needed. Accordingly, since the time this analysis was conducted, RNTCP policies and interventions are built to address these factors and are being scaled-up in India. These are designed to detect DR-TB early with universal rapid molecular DST before multiple prolonged treatment exposures or before cavitary disease sets in; to treat all variants of DR-TB promptly with regimen designs guided by baseline SL-LPA triage for FQ and/or SLI resistance including shorter MDR-TB regimen (if susceptible to FQ and SLI) or longer DST guided regimen with newer drugs like Bedaquiline; to build systems for real-time DR-TB surveillance, decentralized counseling to promote adherence, nutritional assessment and supplementation to avert weight loss and influence of undernutrition and to prevent further emergence of DR-TB with transition to daily FDC regimen for DS-TB. However, highest political commitment to invest in a robust national strategic plan, systematic vigilant scale up of these new interventions with evidence guided course correction will remain the key to ending TB and DR-TB in India.

## Supporting information

S1 Checklist STROBE Statement—Checklist of items that should be included in reports of cohort studies. (DOC)

S1 Data De-identified data–MDR_TB_Cohort_India–rev. (XLSX)

S1 Fig Flowchart of Interim and Final Outcomes of Initial Cohort of MDR/RR TB patients treated under PMDT services from August 2007 to 31st March 2011 in India. (TIFF)

S2 Fig Smoothed hazard function for missed doses in intensive phase to indicate violation of PH assumption in survival models for time to culture conversion. (TIFF)

S3 Fig Kaplan Meier survival estimates for unfavorable outcomes (a) by BMI.
(TIFF)
