# Supplementary material for: Unacceptable treatment outcomes and associated factors among India's initial cohorts of multidrug-resistant tuberculosis (MDR-TB) patients under the revised national TB control programme (2007–2011): Evidence leading to policy enhancement
Source: PLoS One. 2018 Apr 11;13(4):e0193903. doi: 10.1371/journal.pone.0193903 (PMC5894982; doi:10.1371/journal.pone.0193903)
Supplement: S1 Checklist — (DOC) [file pone.0193903.s001.doc]

STROBE Statement—Checklist of items that should be included in reports of ***cohort studies***

|  | Item No | Recommendation |
| --- | --- | --- |
| **Title and abstract** | 1 | (*a*) Indicate the study’s design with a commonly used term in the title or the abstract |
|  |  | Retrospective cohort analysis of unacceptable treatment outcomes and associated factors among India’s initial cohorts of multi-drug resistant tuberculosis (MDR-TB) patients under revised national TB control programme (2007–2011) |
|  |  | (*b*) Provide in the abstract an informative and balanced summary of what was done and what was found  **What was done:** Clinical and microbiological data abstracted from RNTCP PMDT records were analysed using Cox proportional hazards modelling and adjusted odd’s ratio to evaluate factors associated with initial sputum culture conversion, culture reversion and treatment outcomes as defined by country adaptation of the standard WHO definitions (2008) after controlling for demographic and clinical characteristics.  **What was found:** Of 3712 MDR-TB patients, 1 in 4 patients initially culture converted at a median 100 days, 1 in 5 of those converted had culture reversion and 2 in 3 patient among 2264 (60.9%) patients with treatment outcomes available had unfavourable outcomes. Common factors associated with interim and final outcomes were BMI < 18; ≥ 7 missed doses in IP & CP; cavitary disease; prior treatment episodes weight changes during treatment; males and additional resistance to first line drugs (E,S). In a sub-group of 104 MDR-TB patients, 62 (59.6%) have Ofx resistance. Baseline susceptibility to Ofx and Km significantly doubled and quadrupled the chances for culture conversion respectively while baseline susceptibility to Ofx also significantly reduced the odds of unfavorable treatment outcomes. To address the factors associated with poor treatment outcomes revealed in our study, systematic multi-pronged approach thorough evidenced lead policy enhancement and scale-up is advancing in India. |
| Introduction | | |
| Background/rationale | 2 | Explain the scientific background and rationale for the investigation being reported  India experienced poor treatment outcomes in the early cohorts of the RNTCP from 2007-08 and being home to 1 in 4 of the global MDR-TB patients makes it critical that their treatment outcome and the factors affecting them are systematically evaluated to guide the national programme to take informed decisions on policies and strategies to improve treatment outcomes of subsequent cohorts of patients. |
| Objectives | 3 | State specific objectives, including any prespecified hypotheses  This specific objective was to evaluate microbiological and treatment outcomes along with risk factors for poor outcomes, among the initial cohort of all laboratory-confirmed MDR-TB patients initiated on treatment under RNTCP PMDT services from August 2007 to March 2011. The study also report how the results from this analysis influenced substantial policy changes for PMDT in India. |
| Methods | | |
| Study design | 4 | Present key elements of study design early in the paper  This study is a retrospective cohort analysis based on RNTCP PMDT records. |
| Setting | 5 | Describe the setting, locations, and relevant dates, including periods of recruitment, exposure, follow-up, and data collection  150 districts of 15 states covering 331.5 million with PMDT services available as of March 2011 in India from the study setting.  3172 lab-confirmed MDR-TB patients (including RR-TB patients) from high risk groups put on standard 24-27 month MDR-TB regimen (Km,Ofx,Eto,Cs,E,Z) under the RNTCP from August 2007 to March 2011 were included in the study from 15 DR-TB treatment centers catering to 108 districts. Retrospective data collection from programme records was done from March-June ’12. |
| Participants | 6 | (*a*) Give the eligibility criteria, and the sources and methods of selection of participants. Describe methods of follow-up  There were no predefined exclusion criteria and all eligible MDR-TB patients put on treatment from 15 centres in the defined cohort were enrolled in the study. |
|  |  | (*b*) For matched studies, give matching criteria and number of exposed and unexposed  Since this was the initial cohort of MDR-TB patients in India, there was no possibility of matching in this study. |
| Variables | 7 | Clearly define all outcomes, exposures, predictors, potential confounders, and effect modifiers. Give diagnostic criteria, if applicable  **Independent variables:** Basic demographic characteristics; BMI; initial patient type; prior TB treatment exposure including episodes, duration and source; clinical and microbiological data like co-morbidities (HIV and DM); resistance patterns to all first line drugs (SHRE); grades of chest radiographs and cavitation on chest radiographs; treatment delay by DST method; weight change at 6 and 12 months; treatment adherence (≥7 missed doses during IP & CP) & for a subgroup of patients, baseline second-line DST results for Ofx, Km and Eto was available and considered for analysis.  **Dependent variables:** Initial culture conversion, culture reversion, final treatment outcomes classified as favorable (cured, treatment completed) and unfavorable (deaths, lost to follow up [LTFUs] and treatment failure using standard WHO definitions (2008). |
| Data sources/ measurement | 8* | For each variable of interest, give sources of data and details of methods of assessment (measurement). Describe comparability of assessment methods if there is more than one group  **For Dependent variables:**  Source of data: Treatment registers from the 15 treatment centers.  Assessment or Measurement: Initial culture conversion defined as two consecutive negative culture results more than 30 days apart, culture reversion defined as two consecutive positive culture results more than 30 days apart after conversion and, final treatment outcomes classified as favorable (cured, treatment completed) and unfavorable (deaths, lost to follow up [LTFUs] and treatment failure using standard WHO definitions (2008).  Dates to event from dates to treatment initiation were used to compute time to interim and final outcomes.  **For Independent variables**:  Source of data: The treatment registers, treatment cards and drug-o-gram that detailed history of exposure to past treatment episodes maintained at the 15 treatment centres.  Assessment or Measurement: Nominal variables like Sex, Co-morbidity (HIV, DM), cavitation, initial registration type, previous treatment exposure (source), DST method, resistance to first and second line drugs were measured. Ordinal and series variables like age, age group computed, BMI (computed from baseline weight and height), weight change computed, treatment adherence computed from number of missed doses. Series variables were used to compute median and IQR.  There was not comparable group. |
| Bias | 9 | Describe any efforts to address potential sources of bias  Statistical methods were applied to address potential sources of bias. |
| Study size | 10 | Explain how the study size was arrived at  3712 MDR-TB patients treated under RNTCP PMDT from August 2007 to March 2011 in the 15 selected DR-TB centers formed the study site after excluding patients enrolled from 9 DR-TB centers started recently in early 2011 for which the data required for analysis of earliest interim outcomes were not available as they were very early in their treatment course. |
| Quantitative variables | 11 | Explain how quantitative variables were handled in the analyses. If applicable, describe which groupings were chosen and why  Ordinal and series variables like age, age group computed, BMI (computed from baseline weight and height), weight change computed, treatment adherence computed from number of missed doses. Regression discontinuity model was applied to derive the cut-off for missed doses to dichotomise treatment adherence in IP and CP to analyse it’s optimized impact on treatment outcomes. Series variables were also used to compute median and IQR. |
| Statistical methods | 12 | (*a*) Describe all statistical methods, including those used to control for confounding  Logical approach was applied to statistical analysis with Kaplan Meier cuves (univariate analysis) to test proportional hazard (PH) assumption verified using Schoenfeld residuals from the Cox models across independent variable categories, bivariate Cox model to identify variables followed by multivariate regression model after adjusting for age, sex and HIV status was undertaken. Time dependent hazard ratios were plotted for independent variables that violated the PH assumption. |
|  |  | (*b*) Describe any methods used to examine subgroups and interactions  Detailed in point (a) |
|  |  | (*c*) Explain how missing data were addressed  Missing data were removed from the Cox model. |
|  |  | (*d*) If applicable, explain how loss to follow-up was addressed  Not applicable |
|  |  | (*e*) Describe any sensitivity analyses  Variable treatment adherence (≥ 7 missed doses in IP) with cut-off arrived at using regression discontinuity model. It violated the PH assumption on survival analysis and hence was removed from the multivariate regression model.  Multivariate regression model adjusted for age, sex and HIV status. |
| Results | | |
| Participants | 13* | (a) Report numbers of individuals at each stage of study—eg numbers potentially eligible, examined for eligibility, confirmed eligible, included in the study, completing follow-up, and analysed |
|  |  | (b) Give reasons for non-participation at each stage |
|  |  | (c) Consider use of a flow diagram  Figure 2 details the flow chart that covers point (a) and (b) above |
| Descriptive data | 14* | (a) Give characteristics of study participants (eg demographic, clinical, social) and information on exposures and potential confounders  The characteristics of the study participant are detailed in Table 1 that covers demographic, clinical, social aspects and information on exposure and potential confounders. |
|  |  | (b) Indicate number of participants with missing data for each variable of interest  Figure 2 describes patients whose data was missing due to various reasons in the cascade of treatment.  Data for missing in patients with 1.5% for HIV status, 12.9% for cavitation, 18.5% for DST results to E & S, 23.2% and 35.6% for weight change at 6m and 12m respectively, 3.9% and 11.8% for treatment adherence in IP and CP respectively. Second line DST results missing for only 2.8% (104) of the patients |
|  |  | (c) Summarise follow-up time (eg, average and total amount)  Not applicable |
| Outcome data | 15* | Report numbers of outcome events or summary measures over time  Figure 2 shows 3712 (100%) patients had intial culture conversion and culture reversion information, while 2264 (60.9%) patients had final treatment outcome information available for analysis. |
| Main results | 16 | (*a*) Give unadjusted estimates and, if applicable, confounder-adjusted estimates and their precision (eg, 95% confidence interval). Make clear which confounders were adjusted for and why they were included  Table 1 to 7, details unadjusted estimates, confounder-adjusted estimates and their precision with clear footnotes on which variables were adjusted for. |
|  |  | (*b*) Report category boundaries when continuous variables were categorized  Age group (<15, 15-44, 45-64, >64), BMI (<18, ≥18), Treatment adherence in IP and CP (<7, ≥ 7 missed doses) |
|  |  | (*c*) If relevant, consider translating estimates of relative risk into absolute risk for a meaningful time period  Estimates presented as hazard ratio and adjusted odd ratio |
| Other analyses | 17 | Report other analyses done—eg analyses of subgroups and interactions, and sensitivity analyses  Subgroup analysis to study the association of second line DST results on interim and final treatment outcomes was done and  Sensitivity analysis detailed in 12 (e) above. |
| Discussion | | |
| Key results | 18 | Summarise key results with reference to study objectives  Of 3712 MDR-TB patients, 1 in 4 patients initially culture converted at a median 100 days, 1 in 5 of those converted had culture reversion and 2 in 3 patient among 2264 (60.9%) patients with treatment outcomes available had unfavourable outcomes. Common factors associated with interim and final outcomes were BMI < 18; ≥ 7 missed doses in IP & CP; cavitary disease; prior treatment episodes weight changes during treatment; males and additional resistance to first line drugs (E,S). In a sub-group of 104 MDR-TB patients, 62 (59.6%) have Ofx resistance. Baseline susceptibility to Ofx and Km significantly doubled and quadrupled the chances for culture conversion respectively while baseline susceptibility to Ofx also significantly reduced the odds of unfavorable treatment outcomes. |
| Limitations | 19 | Discuss limitations of the study, taking into account sources of potential bias or imprecision. Discuss both direction and magnitude of any potential bias  Limitations include no data on other potential factors in publish studies impacting treatment outcomes like ADR, cost incurred etc. due to restricted by data extracted from programme records, grades of chest x-ray classification had high inter-reader variability, SLDST data was available in a very small subgroup due to limited SLDST lab capacity, relapse surveillance was not possible. |
| Interpretation | 20 | Give a cautious overall interpretation of results considering objectives, limitations, multiplicity of analyses, results from similar studies, and other relevant evidence  With poor treatment outcomes in initial MDR-TB cohort of patients, India needs to address risk factors identified in this study through bold policy and rapid scale up of plans with interventions like universal DST (using Xpert) guided treatment to address delays in diagnosis and differential treatment regimen for all DR-TB patterns, Shorter MDR-TB regimen or Newer drugs containing regimen based on baseline SL-LPA Ofx/KM resistance, counselling, nutritional support, ICT enabled treatment adherence monitoring, DR-TB surveillance and innovations to end TB and DR-TB in India. |
| Generalisability | 21 | Discuss the generalisability (external validity) of the study results  Although the study results gave specific strategic measures to address factors associated with interim and final outcomes, as the data was representing MDR-TB patients detected from a highly selected risk group only from 150 districts, generalizability of results required ongoing periodic analysis of the programme data, in spite of the fact that ~25% of the data was externally and physically validated by a team of WHO consultants. |
| Other information | | |
| Funding | 22 | Give the source of funding and the role of the funders for the present study and, if applicable, for the original study on which the present article is based  None |

*Give information separately for exposed and unexposed groups.

**Note:** An Explanation and Elaboration article discusses each checklist item and gives methodological background and published examples of transparent reporting. The STROBE checklist is best used in conjunction with this article (freely available on the Web sites of PLoS Medicine at http://www.plosmedicine.org/, Annals of Internal Medicine at http://www.annals.org/, and Epidemiology at http://www.epidem.com/). Information on the STROBE Initiative is available at http://www.strobe-statement.org.
